# Evolution of trust and trustworthiness: social awareness favours personality differences

**DOI:** 10.1098/rspb.2008.1182

**Published:** 2008-10-28

**Authors:** John M. McNamara, Philip A. Stephens, Sasha R.X. Dall, Alasdair I. Houston

**Affiliations:** 1 Department of Mathematics, University of Bristol Bristol BS8 1TW, UK; 2 School of Biosciences, University of Exeter Cornwall Campus, Penryn TR10 9EZ, UK; 3 School of Biological Sciences, University of Bristol Bristol BS8 1UG, UK

**Keywords:** personality differences, cooperation, evolution, trust, game theory, behavioural syndromes

## Abstract

Interest in the evolution and maintenance of personality is burgeoning. Individuals of diverse animal species differ in their aggressiveness, fearfulness, sociability and activity. Strong trade-offs, mutation–selection balance, spatio-temporal fluctuations in selection, frequency dependence and good-genes mate choice are invoked to explain heritable personality variation, yet for continuous behavioural traits, it remains unclear which selective force is likely to maintain distinct polymorphisms. Using a model of trust and cooperation, we show how allowing individuals to monitor each other's cooperative tendencies, at a cost, can select for heritable polymorphisms in trustworthiness. This variation, in turn, favours costly ‘social awareness’ in some individuals. Feedback of this sort can explain the individual differences in trust and trustworthiness so often documented by economists in experimental public goods games across a range of cultures. Our work adds to growing evidence that evolutionary game theorists can no longer afford to ignore the importance of real world inter-individual variation in their models.

## 1. Introduction

It is increasingly evident that individuals of a diverse range of species show consistent differences in their behaviour, even under standardized conditions (Wilson *et al.* 1994; Wilson 1998; Budaev *et al.* 1999*a*,*b*; Gosling & John 1999; Fischbacher *et al.* 2001; Gosling 2001; Sih *et al.* 2004*b*). Such ‘personality types’ (Pervin & John 1999) may be stable across contexts, e.g. an individual that is aggressive towards conspecifics may also be bolder in exploring novel environments; Dingemanse & Reale 2005*a*) and/or over time within a single context, e.g. in the presence of a potential predator, individuals may show consistent flight reactions over long periods of time (Boissy 1995; Sih *et al.* 2004*b*). Interest in the evolution and maintenance of such behavioural variation is burgeoning (Macdonald 1995; Dall *et al.* 2004; Sih *et al.* 2004*a*; Dingemanse & Reale 2005*b*; Nettle 2005; McElreath & Strimling 2006; Nettle 2006; Reale *et al.* 2007; Stamps 2007; Wolf *et al.* 2007). Recent modelling work (McElreath & Strimling 2006; Wolf *et al.* 2007) has focused on potential adaptive explanations of consistency across contexts. Here, by contrast, we assume individual differences that are stable over time, and explore the evolutionary consequences of such personality differences within a particular context. Our aim is to identify a selective force that can maintain a range of such personalities within the same population. Specifically, in a cooperative context, we are interested in how selection can prevent all interacting individuals evolving towards the same monomorphic optimum.

Evolutionary game theory shows that, in principle, frequency-dependent selection can maintain a range of trait values within the same population. But the crucial question is often what biological factor (or factors) is likely to generate the requisite frequency-dependent effects? Here, we offer a novel perspective on this question. Put succinctly, we show that natural variation in a social context can itself promote frequency dependence. In other words, variation provides the necessary selection pressure to generate variation.

Within evolutionary game theory, the traditional approach focuses on the mean values of continuous traits. The implication is that this will approximate reality when the variance in trait values is small. However, this ignores the fact that in real populations traits often exhibit substantial levels of variation. In social contexts, once variation is non-negligible, there can be a need to be socially aware, and once individuals are socially aware this changes the selection pressure on all behavioural traits. The resulting evolutionary outcome is then likely to be totally different from that predicted by the traditional approach (McNamara *et al.* 2004). Here, we provide an example in which some individuals are socially aware at evolutionary stability. This results in disruptive selection on the continuous trait being monitored socially. The resultant variation in this trait in turn provides the need for social awareness.

Our focus on a cooperative context is motivated by evidence from experimental economics that people from many cultural backgrounds show consistent differences in their strategic approaches to cooperative economic games, with subjects often exhibiting a range of strategies from completely trusting and trustworthy to tactical cooperation and free riding (Fischbacher *et al.* 2001; Fehr & Fischbacher 2003; Henrich *et al.* 2005; Kurzban & Houser 2005). Indeed, individual differences in neural activity in brain areas associated with reward processing during altruistic giving (Harbaugh *et al.* 2007) and punishment (de Quervain *et al.* 2004) are also being documented. This diversity is particularly striking since traditional game theoretic analyses of cooperation between non-relatives, such as the Prisoner's Dilemma (Axelrod & Hamilton 1981), typically predict outcomes that lack inter-individual variation in cooperative tendencies (but see Boyd *et al.* 2003). Our analysis therefore offers a novel adaptive explanation for real world variation in a key human feature.

### (a) Social awareness in a game of trust and cooperation

**Figure 1:**
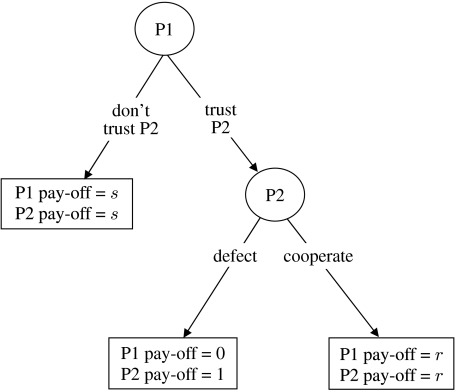
Decision tree for the trust and cooperation game in the simple version (without sampling), showing pathways and outcomes contingent on the behaviours of individuals adopting the role of P1 and individuals adopting the role of P2.

We illustrate our general thesis using a variant of the two-player game of Guth & Kliemt (2000). This game provides a convenient framework for analysing the evolution of trust and cooperation. Pairwise interactions proceed in two phases (figure 1). One individual, chosen at random, is assigned to the role of player one (P1), while the other is assigned to the role of player two (P2). In the first phase, P1 decides whether to trust P2. If P2 is not trusted, both individuals receive a reward *s*, the non-cooperator's pay-off. If P2 is trusted, the game moves to a second phase in which P2 decides whether to cooperate or not (i.e. defect). If P2 cooperates, both individuals receive the cooperator's pay-off *r*. If P2 does not cooperate, P2 receives a pay-off of 1, while P1 gets nothing. Reward magnitudes satisfy 0<*s*<*r*<1.

When P1 has no information about P2 (e.g. individuals only ever interact once), this game has a simple evolutionarily stable outcome. If trusted, it is best for P2 to defect. If P2 will defect P1 does best not to trust P2. Thus, at evolutionary stability, P1 never trusts P2 and both players get pay-off *s*; had they been trusting and cooperative, they would both have received the higher pay-off, *r*. This game can be regarded as a variant of the Prisoner's Dilemma game (Axelrod & Hamilton 1981).

In our extension of this game, we allow P1 to gain information about P2, and let the frequencies of behavioural types evolve as frequency-dependent responses to each other. We make three principal changes to the basic model analysed elsewhere (Guth & Kliemt 2000; McNamara & Houston 2002).

- Previous formulations (McNamara & Houston 2002) considered the unrealistic case where P2 always cooperated or always defected. Typically, however, heritable behavioural traits are continuously distributed within populations (Dall *et al.* 2004; Dingemanse *et al.* 2004; van Oers *et al.* 2005; Blumstein *et al.* 2006; Penke *et al.* 2007). To reflect this, we model an individual's heritable (unconditional) tendency to cooperate in role P2 as specified by *p* (0≤*p*≤1), where *p* is the probability of cooperating.
- To highlight the importance of social awareness, P1 individuals have the option of obtaining information on P2s at a cost. In our specific model, this information is observed by sampling; we allow P1 to observe *n* previous P2 decisions by the individuals playing P2 and base their decision on what they observe. Specifically, the heritable trait of P1s is their tendency to accept P2s in phase 1 of the game. They may be unconditional accepters (UA; always accepting P2 without sampling), unconditional rejecters (UR; always rejecting P2 without sampling) or one of *n* sampling types. The sampling types are specified by an integer *k* where 1≤*k*≤*n*. Type *k* samplers accept the P2 if and only if the P2 was trustworthy on at least *k* of the *n* occasions. Samplers pay a cost *c* (0≤*c*<*s*) reflecting, for example, the costs of using and maintaining the cognitive machinery required to keep track of the behaviour of others (Stephens 2007). Unconditional strategies do not pay a sampling cost. Completely consistent (UA, UR) and/or less stable (type *k* samplers) individual patterns of P1 trust are free to evolve in our formulation.
- Mutation is a ubiquitous source of trait variation in biological systems and can have unexpected effects on the direction of selection (McNamara *et al.* 2004, 2008) so we allow for both P1 and P2 traits to be inherited with mutation.

## 2. Material and methods

We model an infinite population of actors playing the asymmetric game outlined in figure 1. Each individual carries genes specifying behaviour in each of its two possible roles. In each role an individual receives a pay-off that depends on its trait in this role. This pay-off equals the mean outcome of all interactions with other members of the population when in that role; essentially we assume that in each generation, each individual interacts with many other individuals chosen at random. The fitness of an individual equals the sum of its pay-offs in the two roles. Note, however, that since the pay-off in one role does not depend on the pay-off in the other role, at evolutionary stability the trait values in one role are statistically independent of the trait values in the other role. This means that when we track evolution to find an evolutionarily stable strategy, we do not need to keep track of the association between the genes controlling the P1 trait and the genes controlling the P2 trait. Instead, we can just keep track of the distribution of the P1 trait and the distribution of the P2 trait.

Behaviour in the P1 role is controlled by trait 1, defined as either unconditional rejecters (UR), unconditional accepters (UA) or type *k* samplers (1≤*k*≤*n*), where *n* is a constant. For ease of notation, we refer to all possible P1 types by their associated *k* trait value. In particular, URs are assumed to have a trait value of *k*=*n*+1 (i.e. they will never sample or cooperate, because a P2 can never be observed to be trustworthy *n*+1 times out of *n* trials), while UAs are assumed to have a trait value of *k*=0 (i.e. they will always cooperate without sampling because, out of *n* trials, the number of observations of a P2 being trustworthy will always be ≥0). Trait 1 value *k* occurs in the population with frequency *f*_1_(*k*), where $\sum_{k=0}^{n+1}f_{1}(k)=1$.

P2 behaviour is controlled by trait 2, conceptualized as a continuum of values, *p*, in the range 0≤*p*≤1, to capture the continuous nature of such an unconditional behavioural trait. However, for computational purposes, we represent *p* on a fine discrete grid; *p*=0, 0.01, 0.02, …, 0.99, 1. Trait 2 value *p* occurs in the population with frequency *f*_2_(*p*), where $\sum_{p}f_{2}(p)=1$. Evolution of the two traits is not directly linked (except through frequency dependence).

We start with some initial frequency distribution for both traits and iterate one generation at a time. In each generation, new frequencies of each trait value for both traits 1 and 2 are calculated as detailed below. The model continues until stable distributions of frequencies are reached (determined when summed absolute changes, *Δ*, fall below a predefined tolerance; all results reported here used a tolerance of 10^−9^).

### (a) Trait fitness

Pay-offs resulting from dyadic interactions are illustrated in figure 1. For unconditional trait 1 values (*k*=0 and *k*=*n*+1) P1 does not assess P2's previous behaviour and so pays no assessment cost (*c*=0). In all other situations (1≤*k*≤*n*), P1 pays the assessment cost, *c* (where 0<*c*<*s*). So far, for clarity, we have described strategic interactions as particular outcomes within a stochastic framework. Nevertheless, to gain general insight into the evolutionary implications of our logic, we analyse expected outcomes in an infinite population as follows.

The probability that P1 trusts P2 is given by

(2.1) $$a(k,p)=\sum_{x=k}^{n}\frac{n!}{x!(n-x)!}p^{x}{\left(1-p\right)}^{n-x}\;\text{for}\;1\leq k\leq n,$$

with a(0,p)=1 and a(n+1,p)=0.

Equation (2.1) arises because P2 behaviour in interactions is a binomial process (they can cooperate or defect). The term within the summation reflects this, showing the binomial probability that P2 is seen to cooperate *x* times in *n* trials. This is summed for all *x*≥*k*.

The mean pay-off to P1 for a random interaction is given by

(2.2a) $$w_{1}(k)=\sum_{p}f_{2}(p)\left\{\left[1-a(k,p)\right]s+a(k,p)pr\right\}-c\;\text{for}\;1\leq k\leq n,\;\text{or},$$

(2.2b) $$w_{1}(k)=\sum_{p}f_{2}(p)\left\{\left[1-a(k,p)\right]s+a(k,p)pr\right\}\;\text{otherwise}.$$

Equations (2.2*a*) and (2.2*b*) differ only because samplers are assumed to pay a cost of sampling, *c*. Otherwise, both formulations show (within braces) that the expected reward of an interaction with a given type of P2 is the non-cooperator's pay-off, *s*, multiplied by the probability of not trusting P2, 1−*a*(*k,p*), plus the probability of trusting P2, *a*(*k,p*), multiplied by the pay-off from doing so, *pr*. This is summed over all possible P2 types that the P1 can encounter, weighted by the probability of such an encounter.

For a P2 with trait 2 value *p*, the mean pay-off from an interaction with a random actor is given by

(2.3) $$w_{2}(p)=\sum_{k=0}^{n+1}f_{1}(k)\left\{\left[1-a(k,p)\right]s+a(k,p)\left[pr+(1-p)\right]\right\}.$$

Equation (2.3) is similar to the pay-offs for P1s. Within the braces, the first term shows the probability that the P1 does not trust, multiplied by the non-cooperator's pay-off, *s*. The second term shows the probability that the P2 is trusted, multiplied by the pay-off to the P2 from such an interaction. The latter pay-off has two components: either P2 cooperates (with probability *p*), in which case the pay-off is *r*, or P2 defects (with probability 1−*p*), in which case the pay-off is 1. Again, the pay-offs are summed for all possible P1 types that can be encountered, weighted by the probability of such encounters.

### (b) Changing trait frequencies

**Figure 2:**
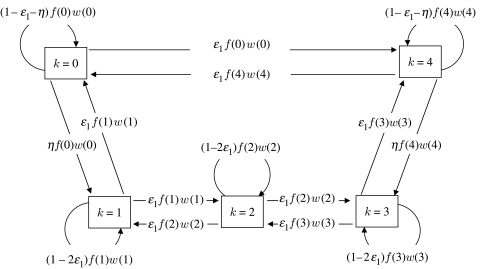
Flow diagram illustrating the source of recruitment to each P1 trait value in the *n*=3 case (corresponds to equation (2.7) in the main text). Note that mutation between similar types (i.e. between unconditional traits or between conditional traits) occurs at the rate *ϵ*_1_. Mutation from conditional to unconditional types also occurs at that rate. By contrast, mutation from unconditional to conditional types is assumed to occur at a lower rate, *η*, where *η*≤0.5*ϵ*_1_ (see text for further details), reflecting the lower likelihood of the more complex, sampling strategies arising.

Mutation rates in the model are controlled by three separate parameters (figure 2). For P1s, mutation from URs to UAs (and vice versa), from *k*=1 samplers to UAs, from *k*=*n* samplers to URs, and between *k*=*i* samplers and *k*=*i*+1 samplers (and vice versa), occurs at the rate *ε*_1_ in each generation. To represent lower rates of mutation from unconditional strategies to the more sophisticated sampler strategies, mutation from UAs to *k*=1 samplers and from URs to *k*=*n* samplers, occurs at a lower rate *η* (*η*≤*ε*_1_/2). This seems biologically realistic, since the more sophisticated samplers may be less likely to arise by chance from the unconditional acceptors or rejecters—for instance, the origin of conditionality may require relatively more mutational steps than switching from one unconditional action to another (or varying levels of scepticism) because the ability to elicit both actions as well as process information must be acquired. Using a mutation rate from unconditional to conditional strategies that is lower than that between other pairs of P1 traits i.e. *(η*<ϵ_1_) does not increase the frequency with which disruptive selection occurs on the P2 trait). However, it does emphasize that disruptive selection is a consequence of genuine selection for conditional P1 traits, rather than mutation to those traits alone. Indeed, several variant sets of assumptions regarding mutation on the P1 trait were examined (including uniform mutation rates between conditional and unconditional traits, and potential mutation between all trait values); all variants produced the general effects that we report here. Finally, P2 mutation occurs between neighbouring trait values on the grid of values at the rate *ε*_2_.

For unconditional P1 trait values, recruitment, *R*(*k*), is given by

(2.4) $$R_{1}(k)=\{\begin{array}{ll} (1-\epsilon_{1}-\eta )f_{1}(0)w_{1}(0)+\epsilon_{1}f_{1}(1)w_{1}(1)+\epsilon_{1}f_{1}(n+1)w_{1}(n+1), & k=0,\\ (1-\epsilon_{1}-\eta )f_{1}(n+1)w_{1}(n+1)+\epsilon_{1}f_{1}(n)w_{1}(n)+\epsilon_{1}f_{1}(0)w_{1}(0), & k=n+1. \end{array}$$

Here, the total recruits produced by individuals bearing any trait value are given by the frequency of that trait value multiplied by its fitness. Total recruitment in either case is the sum of recruits from three sources, corresponding to the three terms: from individuals with the focal trait (subtracting *ϵ*_1_+*η* that mutate away from that trait); from individuals with the neighbouring trait (including only the *ϵ*_1_ recruits that mutate to the focal trait); and from the other unconditional strategy (again, including only the *ϵ*_1_ recruits that mutate to the focal trait). Note that for *n*=0, *η*=0 and the second term in each case in equation (2.4) is omitted.

For samplers (occurring only when *n*>0), the situation is slightly more complicated. Specifically, if *n*=1, recruitment is given by

(2.5) $$R_{1}(k)=(1-2\epsilon_{1})f_{1}(1)w_{1}(1)+\eta f_{1}(0)w_{1}(0)+\eta f_{1}(n+1)w_{1}(n+1).$$

Here, the first term corresponds to recruitment from the focal trait (subtracting the 2*ϵ*_1_ recruits that mutate away from that trait). The second and third terms correspond to low levels of recruitment arising from mutation in recruits of the two unconditional strategies. When *n*=2, recruitment is

(2.6) $$R_{1}(k)=\{\begin{array}{ll} (1-2\epsilon_{1})f_{1}(1)w_{1}(1)+\eta f_{1}(0)w_{1}(0)+\epsilon_{1}f_{1}(2)w_{1}(2), & k=1,\\ (1-2\epsilon_{1})f_{1}(2)w_{1}(2)+\eta f_{1}(n+1)w_{1}(n+1)+\epsilon_{1}f_{1}(1)w_{1}(1), & k=2. \end{array}$$

Finally, for *n*≥3, recruitment is given by

(2.7) $$R_{1}(k)=\{\begin{array}{ll} \begin{array}{l} \left(1-2\epsilon_{1})f_{1}(1)w_{1}(1)+\eta f_{1}(0)w_{1}(0\right)\\ +\epsilon_{1}f_{1}(2)w_{1}(2), \end{array} & k=1,\\ \begin{array}{l} \left(1-2\epsilon_{1})f_{1}(k)w_{1}(k)+\epsilon_{1}f_{1}(k-1)w_{1}(k-1\right)\\ +\epsilon_{1}f_{1}(k+1)w_{1}(k+1), \end{array} & 1<k<n,\\ \begin{array}{l} \left(1-2\epsilon_{1})f_{1}(n)w_{1}(n)+\eta f_{1}(n+1)w_{1}(n+1\right)\\ +\epsilon_{1}f_{1}(n-1)w_{1}(n-1), \end{array} & k=n. \end{array}$$

For clarity, this more complex situation is illustrated in figure 2.

The frequency of individuals carrying trait value *k* in the next generation is then calculated as

(2.8) $$f_{1}^{\prime }(k)=\frac{R_{1}(k)}{\sum_{k=0}^{n+1}R_{1}(k)}.$$

The process of calculating changes in the frequencies of values for trait 2 is similar, as follows. Recall that trait 2 is modelled as discrete, with potential values separated by the interval *i*=0.01 (i.e. P2 traits had 101 possible values). First, recruitment is calculated by

(2.9) $$R_{2}(p)=\{\begin{array}{ll} (1-\epsilon_{2})f_{2}(0)w_{2}(0)+\epsilon_{2}f_{2}(i)w_{2}(i), & p=0,\\ (1-2\epsilon_{2})f_{2}(p)w_{2}(p)+\epsilon_{2}f_{2}(p-i)w_{2}(p-i)+\epsilon_{2}f_{2}(p+i)w_{2}(p+i), & 0<p<1,\\ (1-\epsilon_{2})f_{2}(1)w_{2}(1)+\epsilon_{2}f_{2}(1-i)w_{2}(1-i), & p=1. \end{array}$$

The frequency of individuals carrying trait 2 value *p* in the next generation is then calculated as

(2.10) $$f_{2}^{\prime }(p)=\frac{R_{2}(p)}{\sum_{p}R_{2}(p)}.$$

### (c) Assessing stability

For some parameter sets stable solutions could not be found, even after running simulations for very long time frames (greater than 10^7^ generations). Typically, simulations that failed to stabilize were characterized by fluctuations in the summed absolute changes of trait frequencies, *Δ*, with no downward trend in that value. Consequently, all simulations that failed to stabilize were terminated after 10^7^ generations or after 50 000 changes in the direction of magnitude of *Δ* (recorded following the first 10^5^ generations). Extensive computations revealed that results were entirely robust to initial conditions (i.e. initial frequency distributions on the two traits).

## 3. Results and discussion

**Figure 3:**
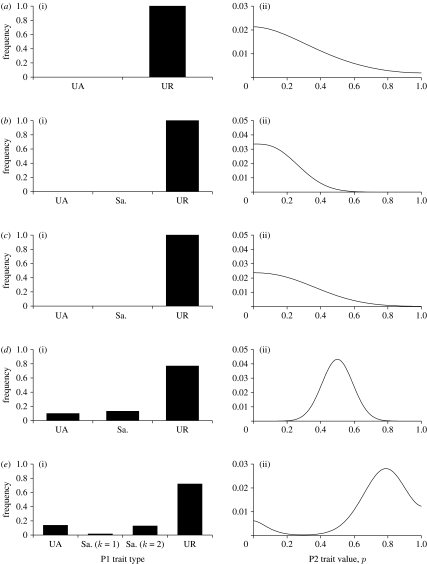
Example outcomes from the asymmetric trust and cooperation game. (*a*(i),(ii)) *n*=0 and sampling is thus not possible. Even with high mutation on the P2 trait (*ε*_2_=0.1) the modal value of *p* is always zero. Other parameters: *s*=0.3; *r*=0.6; *ε*_1_=0.0001. (*b*–*d*) *n*=1, permitting some samplers (denoted Sa.). In each case, *s*=0.3; *r*=0.6; *c*=0.005; *ε*_1_=0.0001; *η*=0.00001. Mutation on the P2 trait is increasing: (*b*(i),(ii)) *ε*_2_=0.001; (*c*(i),(ii)) *ε*_2_=0.01; (*d*(i),(ii)) *ε*_2_=0.1. Note that for low and moderate mutation on the P2 trait (*b*,*c*), P1s gain nothing by sampling. However, when P2 mutation is high (*d*), sampling by P1s is worthwhile; the presence of samplers ensures increased trustworthiness among P2s. (*e*(i),(ii)) Example of a stable, bimodal outcome when *n*=2. Parameter values: *s*=0.56; *r*=0.77; *c*=0.04; *ε*_1_=0.001; *η*=0.0004; *ε*_2_=0.08. In this situation, the mixture of P1 traits, which includes samplers, maintains a bimodal distribution of P2 traits. The bimodal distribution of P2 traits maintains the need to sample, and hence maintains the P1 mixture.

To illustrate the crucial role of social awareness in driving polymorphisms in P2 behaviour, consider first the case where no sampling is possible (*n*=0). All P2s do equally well against URs and so *p* can drift. Nevertheless, mutation always ensures the presence of some UAs (which can also increase in frequency if *p* drifts to sufficiently high levels). This favours untrustworthy behaviour because pay-offs for P2s decrease linearly with their increasing *p* in the presence of UAs (box 1); the result is a modal value of *p* at zero (and therefore the presence of UAs is only driven by mutation) as illustrated in figure 3*a*. Thus, it is not possible to maintain reasonable levels of trustworthiness (and trust) without social awareness (Guth & Kliemt 2000; McNamara & Houston 2002).

When sampling is possible (*n*≥1) the presence of samplers selects for some degree of trustworthiness in the P2 trait, while the presence of UAs selects for untrustworthiness (box 1). The relative frequencies of samplers and UAs determine the direction of selection on the P2 trait (recall all P2s do equally well against URs). Thus, even if both samplers and UAs are selected against, the low absolute numbers of samplers maintained by mutation–selection balance can select for trustworthiness in the P2 trait. To avoid this occurring, we set the rate of mutation to sampling types to be much lower than between UAs and URs (see §2). As a consequence, in the results we present below, levels of samplers are maintained through active selection (rather than simply by mutation). In general, if there is little variation in the P2 trait then UAs or URs (or both) have higher pay-offs than samplers (box 1). This is because it is only worth paying the cost of sampling if there is something useful to be learnt by sampling. Thus, at evolutionary stability, sampling is maintained by frequency-dependent selection only if sufficient variation in the P2 trait is maintained.

In the simplest case where sampling is possible (*n*=1) P1s are limited to UAs, samplers with *k*=1 and URs. Extensive computations reveal only unimodal distributions of the P2 trait at evolutionary stability. When the P2 trait mutation rate is low, the variation in this trait is low; selection acts against samplers and the modal value of the P2 trait is zero (figure 3*b*). As the mutation rate increases (figure 3*b*–*d*), the increased variance can mean that it is worth paying the cost of sampling (box 1). When this happens, the direction of selection on the P2 trait changes and the modal value of the P2 trait increases (figure 3*d*).

When opportunities exist for more extended social observation (*n*≥2) a second, novel mechanism can maintain variation in the P2 trait. For example, when *n*=2 a P1 population consisting of a mixture of UAs, URs and samplers (mostly quite sceptical *k*=2 types) and a bimodal P2 population can be evolutionarily stable (figure 3*e*). As stated above, UR individuals have no effect on the direction of selection on the P2 trait. Thus, this direction is determined by the ratio of UAs to samplers. P2s maximize their pay-off in interactions with UAs by being completely untrustworthy (*p*=0). In interactions with samplers the P2 pay-off is maximized at an intermediate value of *p*. This value is a compromise between gaining acceptance through a high *p* value and optimally exploiting P1 once accepted. The mixture of UAs and samplers at evolutionary stability results in P2 fitness being a bimodal function of *p* with two equally high peaks, one involving complete and consistent untrustworthiness (*p*=0) and the other at a positive, but less consistent, level of trustworthiness. Consequently, there is disruptive selection on the P2 trait, and the evolutionarily stable distribution of this trait is bimodal. This bimodal distribution means that there is high variance in the P2 trait, ensuring that sampling is maintained. In other words, the mixture of P1 traits, which includes samplers, maintains a bimodal distribution of P2 traits. The bimodal distribution of P2 traits maintains the need to sample, and hence maintains the P1 mixture.

**Figure 4:**
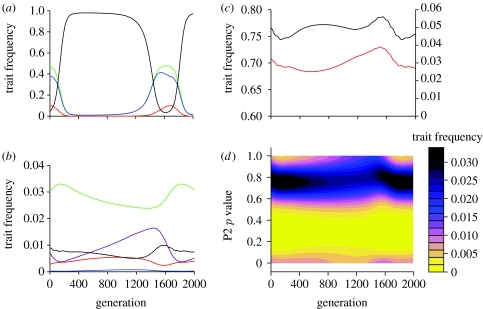
Cyclical dynamics in the asymmetric trust game. (*a*–*d*) An example of cyclical dynamics for *n*=2: (*a*) Frequencies of P1 traits (green curve, UA; orange curve, Sa. (*k*=1); blue curve, Sa. (*k*=2); black curve, UR), (*b*) frequencies of selected P2 traits (violet curve, *p*=1.00; green curve, *p*=0.75; orange curve, *p*=0.50; blue curve, *p*=0.25; black curve, *p*=0.00), (*c*) summary characteristics of trustworthiness in P2s (orange curve, mean *p*; black curve, var (*p*)) and (*d*) contour diagram of fluctuating P2 frequencies (all trait values). Note that, at the start of the time frame, P2s of moderate to high trustworthiness predominate in the population. This promotes the frequency of UAs, in turn selecting for less trustworthy P2s, and reducing the mean trustworthiness in the P2 population (*c*). Decreasing trustworthiness selects for URs and against more cooperative P1s (*a*). Selection on P2s is thus reversed and mean trustworthiness increases, eventually returning the situation to its starting conditions. (*a*–*d*) Shows slightly over one full cycle. Sa., samplers.

**Figure 5:**
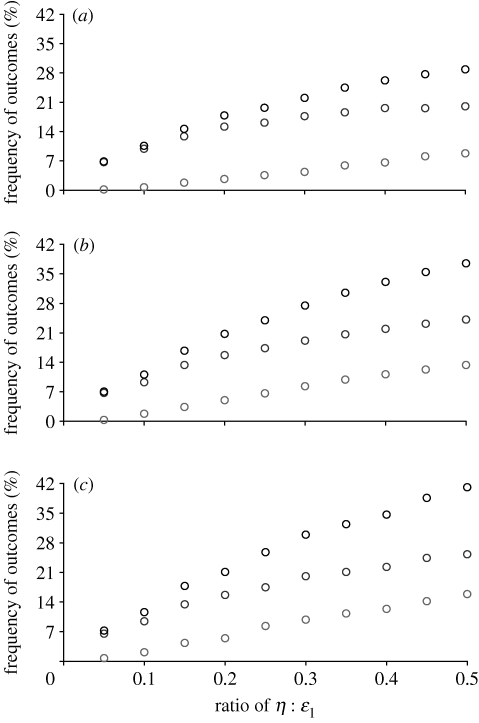
As opportunities for social observation increase (i.e. as *n* increases: (*a*), *n*=2; (*b*), *n*=3; (*c*), *n*=4), so the proportion of parameter space producing stable bimodal (bottom circles) or unstable (middle circles) increases (total bimodal and unstable cases are indicated by the top circles). In each case, parameter space was sampled randomly by selecting 10^5^ parameter sets, each selected in the following order: *s* (0.1<*s*<0.8); *r* (*s*+0.05<*r*<1.0); *c* (0.001<*c*<*s*/2); *ε*_2_ (0.001<ε_2_<0.1); *ε*_1_ (0.0002<*ε*_1_<*ε*_2_/10); *η* (*ε*_1_/100<*η*<*ε*_1_/2).

**Figure 6:**
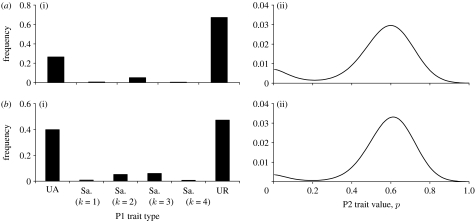
Examples of stable, bimodal outcomes where greater opportunities exist for P1s to monitor the behaviour of P2s. (*a*(i),(ii)) *n*=3; *s*=0.48; *r*=0.88; *c*=0.04; *ε*_1_=0.00045; *η*=0.00021; *ε*_2_=0.08. (*b*(i),(ii)) *n*=4; *s*=0.49; *r*=0.85; *c*=0.03; *ε*_1_=0.00072; *η*=0.00033; *ε*_2_=0.11. Sa., samplers.

Bimodal solutions can either be stable, as in figure 3*e* or maintained as a result of cycling. The forces giving rise to these outcomes are the same. The dynamics maintaining polymorphisms are illustrated in figure 4. Increasing *n* above 2 leads to an increase in the proportion of unstable and bimodal outcomes (figure 5). Examples for *n=*3 and *n=*4 are shown in figure 6.

Our analysis clearly demonstrates how social awareness—trusting on the basis of prior evidence of trustworthy behaviour—can encourage variability in trustworthiness. Such variability in turn favours some socially aware individuals, even when their awareness is costly (Nettle 2006). In our model, individuals can gain information about others by observing their behaviour in the past, with the parameter *n* representing the quality of this information. There is a certain lack of realism in this formulation. In particular, we might expect that in a real population the ease with which P2 can be observed being trusted by others would depend on the number of UAs. In the current model, however, we have chosen not to allow *n* to vary with the proportion of UAs. This is because our general conclusion is not restricted to the specific manner in which information is obtained; it applies to any system in which an individual can gain information about others at a cost. Potential methods of acquiring information include communication of information by third parties (when the cost is in terms of the time needed to interact with others and be part of a social network), and acquiring information by observing facial expression (when the cost is in terms of development of the neural machinery needed to interpret facial expressions). Although we analyse a specific model, our general message—that variation begets variation in social contexts—has broad implications for the analysis of evolutionary games in biology and to a wide range of disciplines that use game theory. Game theory needs to take both variance and social sensitivity into account in a systematic manner if it is to be an effective tool for dealing with real populations and in particular when dealing with the inter-individual variation associated with personality.

Our formulation can also be related to models of indirect reciprocity and the evolution of cooperation (Nowak & Sigmund 1998; Leimar & Hammerstein 2001). Nowak & Sigmund (1998) studied a game in which a donor decides whether to give aid to a recipient. The donor's decision depends on the image score of the recipient. An individual's image score increases when the individual is observed to give aid to another individual and decreases when the individual is observed not giving aid when a donation was possible. In this game, donors should be concerned about their reputation and hence, as Leimar & Hammerstein (2001) pointed out, donors should base their decisions on their own image score rather than on the image score of the recipient. Although our model involves observations and a form of assessment, our pay-off structure differs from that of Nowak & Sigmund. In our game, the pay-off to P1 depends on the accuracy with which P1 assess the personality of P2. It is therefore reasonable for P1 to make decisions on the basis of a score that is assigned to P2. Furthermore, P1 is not observed so there is no pressure on P1 to establish a reputation. These features mean that the objection raised by Leimar and Hammerstein does not apply.

Finally, our work demonstrates how the diversity in trust and trustworthiness so often documented in experimental public goods games (Fischbacher *et al.* 2001; Fehr & Fischbacher 2003; Henrich *et al.* 2005; Kurzban & Houser 2005) can evolve in response to the premiums on selfishness in the presence of trusting individuals (who cannot be bothered to monitor the social interactions going on around them), coupled with some incidence of monitoring effort that such selfishness necessitates. Thus, the ‘arms race between observing and being observed’ (Milinski & Rockenbach 2007) may explain yet another important facet of human altruism and altruistic tendencies.

## Box 1. Variation in P2 behaviour favours P1 samplers and vice versa.

Consider a population where the P2 trait *p* has mean *μ=E*{*p*} and variance *σ*^2^=var(*p*). In this population, the pay-off to an unconditional accepter (UA) is

$$W_{\text{UA}}=E\{pr\}=\mu r,$$

and the pay-off to an unconditional rejecter (UR) is

$$W_{\text{UR}}=s.$$

Thus

$$W_{\text{UA}}>W_{\text{UR}}\Leftrightarrow \mu >s/r.$$

Now suppose that *n*=1. In this case, a sampler accepts a P2 if and only if they are observed to be trustworthy on the one occasion they are observed (*k*=1). Suppose that a P2 has trait value *p*. Then a sampler rejects this P2 (receiving pay-off *s*) with probability 1−*p* and accepts the P2 (receiving expected pay-off *pr*) with probability *p*. Thus, in its interaction with this particular P2, a sampler has expected pay-off

$$w(p)=(1-p)s+p^{2}r-c.$$

The mean pay-off to the sampler is therefore

$$W_{s}=E\{w(p)\}=(1-\mu )s+(\mu^{2}+\sigma^{2})r-c.$$

This formula shows that both the mean and variance of *p* affect the pay-off for sampling. When *μ*=*s*/*r*, so that UAs and URs do equally well, it is easy to see that samplers do better if and only if

$$\sigma^{2}>c/r.$$

For other values of *μ*, the variance (*σ*^2^) needs to be higher still if samplers are to do better than both UAs and URs.

With this population the pay-offs to a P2 player with trait value *p* in an interaction with a UA, a UR, and a sampler are

$$V_{\text{UA}}(p)=1-(1-r)p,$$

$$V_{\text{UR}}(p)=s,$$

and

$$V_{s}(p)=s+(1-s)p-(1-r)p^{3},$$

respectively. Thus pay-off has a maximum at *p*=0 in an interaction with a UA. In an interaction with a sampler, pay-off is maximized at

p=min{1,(1−s)/2(1−r)}.

In particular, it is maximized at an intermediate value of *p* provided 2*r*−*s*<1. This intermediate value of *p* is an optimal compromise: as *p* increases, the probability of being trusted increases, but the pay-off to the P2 (if it is trusted) decreases.
